# Polyphosphazene-Based Nanotherapeutics

**DOI:** 10.3390/jfb16080285

**Published:** 2025-08-02

**Authors:** Sara Gutierrez-Gutierrez, Rocio Mellid-Carballal, Noemi Csaba, Marcos Garcia-Fuentes

**Affiliations:** 1 Department of Pharmacology, Pharmacy and Pharmaceutical Technology, CiMUS Research Centre, University of Santiago de Compostela, 15782 Santiago de Compostela, Spain; sara.gutierrez.gutierrez@rai.usc.es (S.G.-G.); rocio.mellid@rai.usc.es (R.M.-C.); noemi.csaba@usc.es (N.C.); 2 Fundation Institute of Health Research of Santiago (FIDIS), 15706 Santiago de Compostela, Spain

**Keywords:** poly(organo)phosphazenes, PPZs, chemistry, pharmaceutical properties, polyelectrolytic complexes, micelles, polymersomes, drug-conjugates

## Abstract

Poly(organo)phosphazenes (PPZs) are increasingly recognized as versatile biomaterials for drug delivery applications in nanomedicine. Their unique hybrid structure—featuring an inorganic backbone and highly tunable organic side chains—confers exceptional biocompatibility and adaptability. Through precise synthetic methodologies, PPZs can be engineered to exhibit a wide spectrum of functional properties, including the formation of multifunctional nanostructures tailored for specific therapeutic needs. These attributes enable PPZs to address several critical challenges associated with conventional drug delivery systems, such as poor pharmacokinetics and pharmacodynamics. By modulating solubility profiles, enhancing drug stability, enabling targeted delivery, and supporting controlled release, PPZs offer a robust platform for improving therapeutic efficacy and patient outcomes. This review explores the fundamental chemistry, biopharmaceutical characteristics, and biomedical applications of PPZs, particularly emphasizing their role in zero-dimensional nanotherapeutic systems, including various nanoparticle formulations. PPZ-based nanotherapeutics are further examined based on their drug-loading mechanisms, which include electrostatic complexation in polyelectrolytic systems, self-assembly in amphiphilic constructs, and covalent conjugation with active pharmaceutical agents. Together, these strategies underscore the potential of PPZs as a next-generation material for advanced drug delivery platforms.

## 1. Introduction

Nanomedicine applies nanotechnology-based products for specific pharmaceutical interventions to diagnose, treat diseases, and repair damaged tissues. While certain nanosystems can work as therapeutic or imaging agents by themselves, they are mostly applied as carriers to deliver therapeutic molecules to their site of action [1]. Nanotherapeutics offer several advantages for drug delivery, including targeting, enhanced solubility, prolonged half-life, improved therapeutic index, and decreased immunogenicity. These benefits have opened opportunities to introduce molecules with significant therapeutic activity, but poor biopharmaceutical profile, into the first line of treatments [2]. Among the variety of materials studied for nanomedicine, polyphosphazenes have gathered interest due to their unique properties, such as biocompatibility, versatility in chemical modification, and ability to form stable nanostructures. These characteristics make them ideal candidates for constructing nanotherapeutics, enhancing drug delivery efficiency, and improving therapeutic outcomes.

Polyphosphazenes are synthetic polymers constituted by an inorganic backbone of alternating nitrogen–phosphorus atoms linked by resonating single/double bonds [3]. This general structure gives polyphosphazenes high thermal stability, ionic conductivity, and tunable hydrolytic degradability. Furthermore, polyphosphazenes can be customized with various organic, organometallic, or inorganic side groups bound to the phosphorus atom. Those polyphosphazenes where side substituents are organic groups are named poly(organo)phosphazenes (PPZs) [4].

PPZs possess controllable molecular weight, low polydispersity, and multifunctionality through various radicals. Additionally, their degradation leads to non-toxic by-products and controlled release properties, making them highly suitable for diverse clinical applications [5,6]. Numerous studies have demonstrated the tunability of the physicochemical and biological properties of PPZs through the careful selection of substituents. Such tailored PPZ-based materials are promising for designing advanced PPZ-based nanotherapeutics with precise functionalities. Indeed, PPZs have been studied for applications in nanovaccination, drug and gene delivery, and for the fabrication of scaffolds for tissue engineering [6,7,8,9]. The medical relevance of PPZs is showcased by the COBRA-PzF™ coronary stent developed by CeloNova BioSciences, Inc., the first medical device to feature a PPZ coating [10].

To provide added value for nanotherapeutics, PPZs must be rationally designed considering their functionality in the delivery system. Apart from the backbone molecular weight and polydispersity, key considerations include side chain characteristics, such as length, charge, water affinity, and how the resulting structure impacts targeting and biodegradation rates [11,12]. For example, optimal PPZ properties for drug conjugation may include a high polymer molecular weight to prevent renal excretion while keeping fast biodegradation to prevent accumulation within the body. Their metabolites must be harmless and below the renal clearance limit to avoid side effects. PPZs degrade hydrolytically into inorganic phosphate, ammonium salts, and low-molecular-weight derivatives, which are generally biocompatible, especially with amino acid esters or phenoxy groups [13,14,15]. For example, poly[di(carboxylatophenoxy)PPZ] (PCPP) degrades into p-hydroxybenzoic acid, phosphate, and ammonium, none of which are toxic at relevant concentrations [16]. Thus, PPZ metabolism is assumed harmless if side chains are carefully chosen. Still, the side chains’ nature should be considered and toxic thresholds characterized for each PPZ derivative [17].

**Figure 1:**
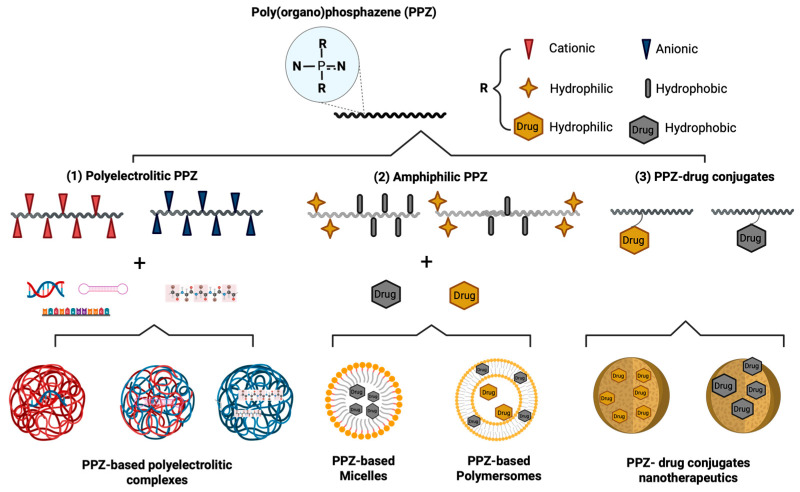
The diagram illustrates the various zero-dimensional PPZ-based therapeutics that can be built depending on the PPZ backbone side groups and the interactions between its subunits and the active molecule. Created in BioRender. Garcia-Fuentes, M. (2025) https://BioRender.com/rhhaqwy (accessed on 29 July 2025). Based on data from references [20,21,22,23,24,25,26,27,28,29,30,31].

In this review, we focus on zero-dimensional (0D) PPZ-based nanotherapeutics. In drug delivery, “zero-dimensional” (0D) means that all three spatial dimensions of the material are within the nanoscale range (always below 1000 nanometers). These materials, often referred to as nanoparticles, possess unique properties due to their size and shape, making them suitable for targeted drug delivery applications [18,19]. In this context, we begin by briefly summarizing the biopharmaceutical characteristics of PPZs, including their chemistry and biomedical properties. Subsequently, PPZ-based nanotherapeutics are classified based on the kind of interactions established with therapeutic molecules (Figure 1): (1) polyelectrolytic PPZs that load drugs through electrostatic interactions, (2) amphiphilic PPZs that encapsulate drugs by hydrophilic and hydrophobic forces, and (3) PPZs conjugated with covalently bonded drugs.

## 2. Chemistry

PPZ structure results in a composition where the organic substituents comprise a very high fraction of the final product, and therefore, they dictate the properties of the final polymers. Two major strategies have been followed for PPZ synthesis. One approach involves a two-step process: first, obtaining a linear polymer and afterward incorporating the organic moieties. The other strategy consists of obtaining the linearized PPZ with the desired side chains in a single step.

### 2.1. PPZ Two-Step Synthesis

#### 2.1.1. Synthesis of Linear Polyphosphazene Precursor

**Figure 2:**
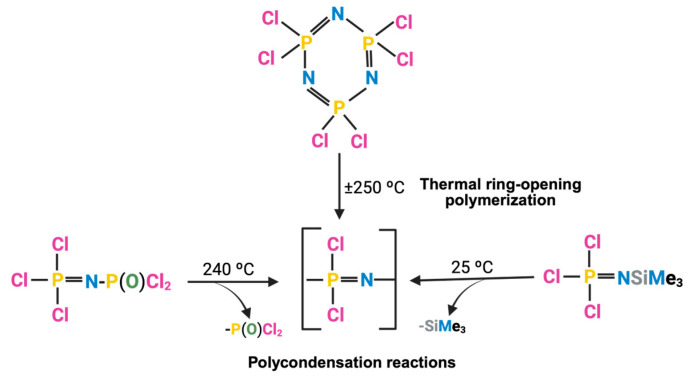
Confluent diagram illustrating various synthesis routes for obtaining the common PPZ precursor PDCP. The downward-pointing vertical arrow represents the thermal ring opening reaction (TROP), while the confluent horizontal arrows in the center represent the two main polycondensation reaction routes. Created in BioRender. Garcia-Fuentes, M. (2025) https://BioRender.com/sx0s4lh (accessed on 29 July 2025).

The main form of linear polyphosphazene is poly(dichloro)phosphazene (PDCP), and this polymer has pivotal importance as a PPZ precursor. The two chlorine atoms of PDCP have high reactivity and enable direct substitution by many organic nucleophiles. This allows the preparation of a great variety of materials starting from this precursor, providing great chemical flexibility. To date, more than 100 derivatives have been prepared starting from PDCP [32]. As previously noted, linear PDCP is overly reactive, which also means that undesirable reactions can occur. PDCP can be obtained through two routes described in the following lines (Figure 2).

Thermal ring-opening polymerization (TROP) is the traditional route to synthesize linear PPZs from the cyclic tetramer polyphosphazenes. Hexachlorocyclotriphosphazene (HCCP) is the most used precursor to produce PDCP following this method. Alternatively, similar cyclophosphazenes bearing bromo or fluor groups can also be used. However, their use is less extended as they require more extreme conditions to open their ring [7,33]. HCCP is converted to PDCP through several steps, but the mechanism of the reaction is still unclear. It is known that the reaction can be performed in molten or solution state, and critical factors for success are high purity of the starting materials, elevated temperatures, and an inert atmosphere. Absolute control of reaction conditions is mandatory; otherwise, the byproduct cross-links and precipitates [34].

Limitations associated with TROP are high PDCP molecular weight, limited reproducibility, broad polydispersity (PDI), and low reaction yields. Moreover, if polymerization conditions are not strictly controlled, chlorine atoms on the phosphorus can react with traces of oxygen or water from the atmosphere and form a rubber-like material, which has no further use [11]. Various catalysts, such as Lewis acids and N-silylated catalysts, have been used to avoid these problems, to control PPZ molecular weight and polydispersity, and to soften reaction conditions, too [33,35].

An alternative route to synthesize PDCP is through polycondensation reactions. One of them is a high-temperature thermolysis of P-trichloro-N-(dichlorophosphoryl) monophosphazene to yield PDCP with a broad molecular weight distribution [36,37]. An improved version of this approach is based on a living cationic polymerization reaction. In these reactions, PDCP is obtained from the polycondensation of a certain number of trichloro(trimethylsilyl)phosphoranimine in the presence of phosphorus pentachloride. In this case, the addition of precursor molecules and polycondensation among them occur sequentially, and a PDCP with a controlled number of subunits can be obtained [38].

Living cationic polymerization occurs at room temperature, which eases the process and ensures that the temperature is not a limiting factor. Nevertheless, the success of the reaction still depends on the purity and concentration of the starting materials and the presence of solvents. When organic solvents are present, the kinetic reactions are accelerated, and those solvents still need to be anhydrous [39,40]. Bulk polymerization with no solvents can also be performed, but this process reduces any control over the molecular weight of the polymer, leading to longer PDCP chains [41].

In summary, living cationic polymerization enables greater control over the reaction. This entails an advantage in obtaining polymers with narrow molecular weight distributions and low polydispersity [42,43]. However, due to side reactions, this method is restricted to short PDCP chains of up to 50–75 subunits [44].

#### 2.1.2. Macro-Substitution

PDCP chlorine atoms can be directly replaced by organic radicals through nucleophilic substitution. Due to the high reactivity of PDCP towards atmospheric components, special care is required for its storage. These storage conditions can be facilitated by stabilizing PDCP through its dilution in diglyme [45]. Although the instability of PDCP poses a challenge for storage, it is advantageous for producing PPZs, as various nucleophiles can easily replace the halogen groups in an aprotic medium, such as that induced by tetrahydrofuran (THF). Consequently, linear polyphosphazenes can be tailored by introducing organic moieties that impart desired properties while maintaining the polymer backbone characteristics, such as chain length and molecular weight distribution [46].

The most common method to produce PPZs is through macromolecular PDCP nucleophilic substitution [17]. This process allows for the introduction of one or more nucleophilic organic side groups in a one-pot or stepwise reaction. Synthesis conditions, such as time and temperature, depend more on the chemical nature of the nucleophiles and the polarity of the reaction medium than on PDCP properties [47]. The reactivity and size of the substituents also affect the reaction yield. Importantly, incomplete substitution may lead to cross-linking and/or degradation of the final polymer chain [48].

**Figure 3:**
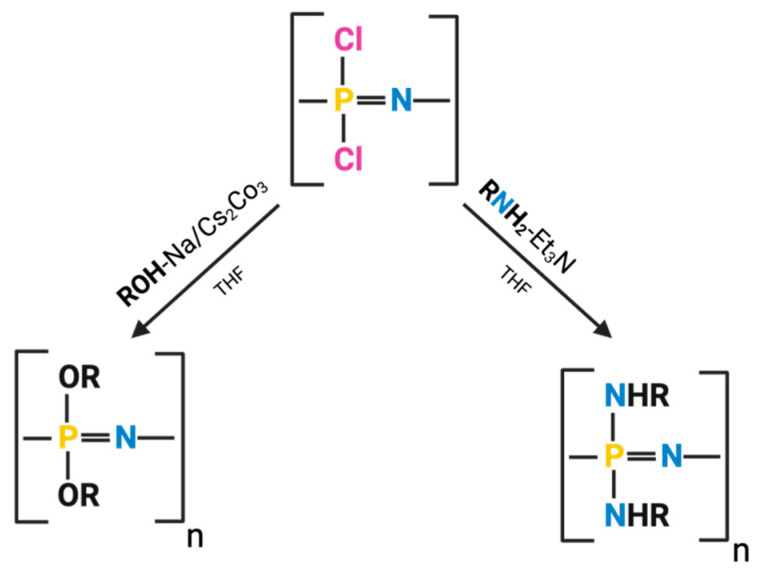
Representative scheme of PDCP macro-substitution in which the chlorine atoms attached to phosphorus are replaced by organic chains ending in hydroxyl groups (reaction represented by the left arrow) or by amino groups (reaction represented by the right arrow). Created in BioRender. Garcia-Fuentes, M. (2025) https://BioRender.com/x57j2mo (accessed on 29 July 2025).

Different types of bonds can form depending on the atom involved in the nucleophilic reaction. In this synthesis method, the main linkages formed between the backbone phosphorus and the nucleophilic groups from the substituents are phosphorus-oxygen bond (P-O) and phosphorus-nitrogen bond (P-N) (Figure 3). Phosphorus-carbon bonding (P-C) is also possible but less common [49].

The main drawback of this macro-substitution reaction is that it cannot be directly applied to molecules with more than one nucleophilic center, as these might attack chlorines from different chains, leading to insoluble crosslinked polymers. To introduce groups with more than one nucleophilic center, protection/deprotection-based synthetic approaches are needed. Alternatively, a second intermediate precursor can be generated, which can then be derivatized to the final material [21,50,51].

### 2.2. One-Step Synthesis

**Figure 4:**
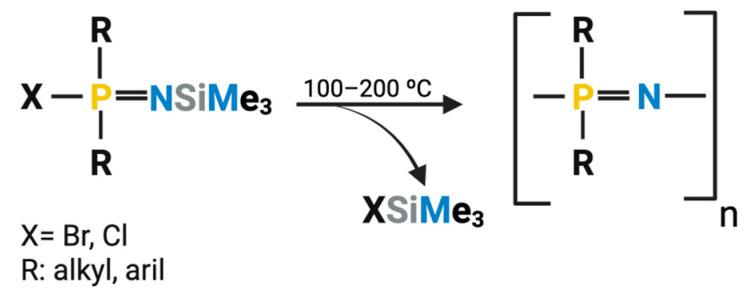
Representative scheme of one-step synthesis in which the PPZ precursor is a phosphoramine already substituted at the phosphorus atom with the final organic side chain. The synthesis of PPZ occurs by polycondensation of monomers of this precursor at high temperatures (reaction represented by the arrow). Created in BioRender. Garcia-Fuentes, M. (2025) https://BioRender.com/zq1kepp (accessed on 29 July 2025).

PPZs can also be synthesized through a direct one-step reaction. In this case, phosphoranimine monomers that are already substituted with organic groups on the phosphorus atom are sequentially added to the reactor and condensed to form the final PPZ (Figure 4) [52]. One-pot synthesis occurs in the absence of solvents and requires elevated temperatures and long reaction times [48]. While this process may appear simpler than two-step synthesis, its complexity lies in the precursor synthesis. This step is crucial to introduce the side chains desired to be present in the final PPZ. Well-established reactions can be used to facilitate the incorporation of specific side groups, particularly P-C bonded organic groups [52]. Still, this method lacks the synthetic flexibility and versatility typical of the divergent PPZ macro-substitution reaction previously described.

One-step synthesis is advantageous when producing polymers that are difficult to obtain through other methods. These include polymers with methyl, ethyl, or phenyl side groups directly linked to the polymer’s skeleton through phosphorus–carbon bonds. There are also limitations to the side groups that can participate in this polymerization; notably, those reagents should not contain functional groups that may interact with the reactive Si-N or P-O bonds from the phosphoranimine precursor [52].

## 3. Key Pharmaceutical Properties

### 3.1. Biodegradability and Tolerability of the Degradation Products

Biodegradability is highly desirable for materials used in the development of pharmaceutical products, such as short-term medical implants, drug delivery systems, and scaffolds for tissue engineering.

Among PPZs, there are many examples of hydrolytically stable polymers. Nevertheless, the versatility of PPZ chemistry allows for the development of water-sensitive materials by incorporating appropriate side groups into the backbone [13]. Based on the groups that impart biodegradability to PPZs, these can be classified into two main categories: aminated polyphosphazenes and alkoxy-substituted polyphosphazenes.

In 1966, Allcock and Kugel reported the first biodegradable aminated PPZ. These aminated PPZs exhibit excellent hydrolytic degradability, and their degradation products are non-toxic. They can be further divided into those substituted with amino acid esters and those substituted with imidazoles. Two mechanisms have been proposed for the hydrolysis of aminated PPZs. In the first, the reaction is triggered by the protonation of atoms in the backbone or the side groups. In the second pathway, the amino residue is removed from the phosphorus atom before the cleavage of the phosphazene ring occurs. Notably, these materials are only hydrolyzed after initially converting the ester or amide units to free carboxylic acid groups, and the mechanisms involved are inhibited in strongly basic media and accelerated in acidic media [53,54].

Alkoxy-substituted polyphosphazenes can be categorized based on their side groups, which may include glyceryl, glycosyl, methyl amino groups, or esters of glycolic or lactic acid. Their degradation mechanisms involve hydrolysis followed by polymer erosion, but the exact pathways remain unclear, and several routes have been proposed [55,56,57].

A significant advantage of biodegradable polyphosphazenes is that their degradation products are typically non-toxic, including phosphate, ammonia, and the byproducts derived from the side groups [11]. The degradation of the backbone into phosphate and ammonia is particularly beneficial compared to other commonly used polymers like poly (lactic-co-glycolic acid) (PLGA), which produce acidic degradation products that can damage encapsulated drugs and tissues. Phosphate and ammonia act as a buffering system, preventing pH fluctuations and avoiding these problems [14,58].

In the case of aminated polyphosphazenes, the degradation products derived from the side groups include the corresponding amine or amino acid [53,54]. For alkoxy-substituted polyphosphazenes, the degradation products depend on the specific side group (Table 1) [15,55,56,57].

The degradation rate of PPZs is influenced by several factors: the stability of their bonds, the water permeability of the polymer matrix, the solubility of the degradation products, and the environmental conditions such as pH and temperature. Modifying the side groups of PPZs to others with different affinities for water can change the water permeability of the polymer matrix [13]. The degradation rate can also be adjusted by varying the side groups’ ratios with different degradability profiles [14]. Among aminated PPZs, those substituted with imidazole are the least stable, and their degradation rate can be reduced by co-substitution with less sensitive groups [53]. Conversely, the degradation rate of amino acid ester-substituted polyphosphazenes can be increased by incorporating side groups with hydrolytically sensitive ester functions, such as amino-acetohydroxamic acid [59]. In another study, Ambrosio et al. developed a mixture of poly(lactide-co-glycolide) with a biodegradable PPZ and observed a moderate degradation rate relative to the individual polymers. Additionally, the degradation byproducts of the PPZ neutralized the acidic degradation byproducts of polyester, thus improving biocompatibility [60].

### 3.2. Stimuli-Responsive Behavior

PPZs can also be modulated to degrade or change their conformation in response to environmental conditions such as temperature, pH, and redox state. Their degradation entails cargo release, so stimuli-responsive PPZ nanotherapeutics are promising candidates for targeted drug delivery. In general, PPZs are somewhat pH-responsive since the protonation of the nucleophilic nitrogen in the backbone eventually triggers polymer degradation and, therefore, cargo release. However, the nature of the side groups must also be considered.

Through the incorporation of pH-sensitive side groups into the PPZ, pH-responsiveness can be increased. Zhou et al. synthesized cross-linked PPZ nanoparticles including doxorubicin and 4-hydroxybenzoic acid (4-hydroxybenzylidene)-hydrazide. The acyl hydrazone bonds in the side groups break under acidic conditions, so these nanosystems remain stable in normal body fluids while they are degraded in acidic environments, such as those found in tumor microenvironment and endosomes [61]. It has been hypothesized that this pH-specific degradation could promote the specific release of doxorubicin only in tumoral cells.

A similar strategy is the design of PPZ conjugates that release the drug in response to pH. Hackl et al. attached ruthenium half-sandwich compounds to branched PPZ by covalent bonds. The amine–ruthenium bonds were hydrolyzed at lower pH values, allowing pH-triggered release of the organometallic compound [62].

The response to pH can also trigger drug release in the endosomes. For example, imidazoquinoline was bound covalently to biodegradable PPZs. Aichhorn et al. showed that those drug conjugates are released at endosomal pH, allowing their interaction with TLR7/8 and, ultimately, their potential as nanovaccines [63].

Reduction-sensitive polymers are typically prepared by incorporating disulfide linkages into macromolecules [64]. Those materials are mostly designed to take advantage of the difference in redox potential between the intracellular and extracellular environment. Indeed, the interior of cells is more reductive due to the overexpression of glutathione. This difference is even more marked in cancer cells, which suggests the potential use of reduction-responsive polymers for targeted release to cancer cells. Hou et al. developed a redox-responsive drug delivery system by the self-framing of doxorubicin and a cysteine derivative into PPZ nanoparticles. Once at the tumor site, glutathione (in its reduced form) disrupted the disulfide bonds, triggering the release of doxorubicin from the nanoparticles [65].

In another context, oxidation-responsive polymers are interesting when high levels of oxidative species are present, such as in inflammatory conditions. Iturmendi et al. substituted PDCP with phenylboronic ester moieties, whose oxidation and cleavage expose the hydrolytically sensitive PPZ backbone, triggering its degradation [66].

Thermo-responsiveness in polymers depends on the presence of a lower critical solution temperature (LCST), above which macromolecules collapse, leading to aggregation or gelation. Achieving the desired LCST is often accomplished by substituting the polymer precursor with amphiphilic oligomers. For instance, substituting with Jeffamines allows tuning of the LCST based on the ratio of ethylene oxide to propylene oxide moieties [67]. Jeffamine-functionalized PPZs exhibit low LCSTs, making them suitable candidates for developing injectable hydrogels. Salinas et al. used Jeffamine-functionalized bottlebrush PPZs to coat mesoporous silica nanoparticles, where the polymers collapsed at body temperature, releasing the nanoparticles’ cargo [68]. The LCST can also be modulated by substituting the PPZ backbone with isopropylacrylamide oligomers, such as poly(N-isopropylacrylamide), and through co-substitution with varying ratios of hydrophobic and hydrophilic substituents [69].

Regarding photo-responsiveness, Iturmendi et al. reported the functionalization of a PPZ backbone with light-sensitive pendant coumarin groups. Upon irradiation, carboxylic acid moieties were exposed, leading to the rapid hydrolysis of the PPZ [70]. Additionally, the structural flexibility of PPZs allows for direct conjugation of photosensitizers, such as hypericin, with potential applications in photodynamic therapy [71].

Stimuli-responsive PPZs can also confer these properties to other systems. For example, Couffin-Hoarau et al. created stimuli-responsive liposomes by anchoring temperature- and pH-sensitive PPZs into phospholipid bilayers [72].

## 4. PPZ-Based Nanosystems

### 4.1. Nanocarriers Based on Polyelectrolytic Complexation

Polyelectrolytic polymers have chemical groups that are charged within the physiological pH range. This charge allows the formation of ionic bonds with oppositely charged molecules, and such interactions can result in the formation of polyelectrolytic complexes (PECs) [73]. Nanometric PECs have been used to protect active molecules, modulate drug pharmacokinetics, and improve transport through biological barriers [74].

PECs can be formed by both natural and synthetic cationic or anionic polymers. By interacting with the active molecule, polyelectrolytic polymers build matrix-type systems whose structural integrity is maintained through electrostatic interactions [75]. Notably, PECs efficiently associate with various drugs, proteins, polynucleotides, and even cells and microorganisms. The conditions of PEC formation are mild and biologically compatible; thus, they have been extensively studied to encapsulate labile macromolecules and cells. PECs exhibit the capability to modulate drug release by three different mechanisms: ionic binding, diffusion within the polymer matrix, and/or matrix degradation mechanisms [76].

**Figure 5:**
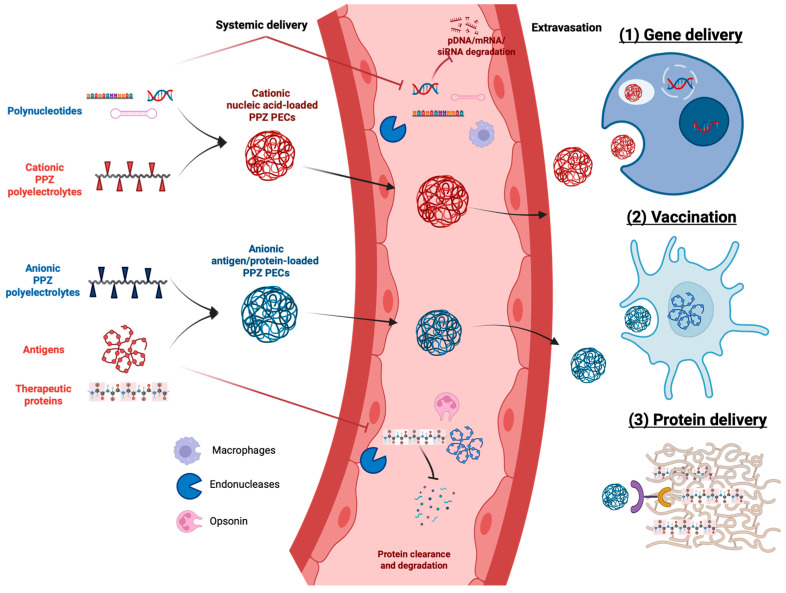
Schematic representation of the association between PPZ polyelectrolytes and oppositely charged therapeutic molecules, along with the therapeutic applications of the resulting PPZ PECs for (1) gene delivery, (2) vaccination, and (3) protein delivery. Created in BioRender. Garcia-Fuentes, M. (2025) https://BioRender.com/lz0qapt (accessed on 29 July 2025). Based on data from references [22,82,83,84,85,86].

Polyelectrolytic PPZs have been synthesized and used to prepare PECs for several drug delivery applications [21,77,78,79,80] (Table 2). Like other cationic polymers, PPZs bearing amine-derived branches have found most of their applications in gene delivery [81]. However, applications of PPZ-based PECs have not been restricted to this area. For example, anionic PPZs have found key applications in vaccine and protein delivery (Figure 5) [77,80].

**Table 2:** PPZ-based nanocarriers built by electrostatic forces. Compilation of experimental studies from the last 10 years (2015–2025) in which cationic and anionic PPZ polyelectrolytes were used for the delivery of oppositely charged therapeutic molecules.

Therapeutic Application	PPZ Derivative	Cargo	In Vitro/In Vivo Model	Main Finding and Ref.
Gene delivery	Imidazole/DMAEA-PPZ	pDNA	In vitro (293T)	Lower toxicity than DMAEA-PPZ [20]
Gene delivery	Cysteamine-PPZ/ Mercaptohexanoic-PPZ	pDNA/siRNA	In vitro (U87) In vivo (U87)	High transfection; low toxicity; antitumor effect in vitro/in vivo [21]
Gene delivery	Cationic/aliphatic-PPZ Mercaptohexanoic acid-PPZ	pDNA (pBMP4)	In vitro (U87/U251), In vivo (U87)	Improved gene transfer in vitro and a potent antitumoral effect in vivo [22]
Vaccine adjuvant	PCPP/PCEP	Various proteins	In vitro (protein solution)	Strong antigen binding. Induces dendritic cells maturation and Th2 cytokine production [87,88]
Vaccine adjuvant	PCPP	EBOV glycoprotein	In vivo (mice)	Efficient immunization through MN-patches and IM administration in mice [89]
Vaccine adjuvant	PCPP	ogp160	Phase I clinical trial	Higher T-cell proliferation than alum-adjuvanted [90]
Vaccine adjuvant	PCPP/PCEP	E2	In vivo (mice)	PCEP PECs produced higher neutralizing antibody titers than Addavax, alum, and PCPP PECs [91]
Vaccine adjuvant	PCEP	H1N1	In vivo (pig)	Strong anti-H1N1 immunogenicity; high IFN-γ, IL-13, IL-17A; no cross-protection against H3N2 [86]
Vaccine adjuvant	PCMP	VLP	In vitro In vivo (mice)	Greater stability and neutralizing titers than alum or Gardasil-9 [92]
Protein delivery	PEGylated-PPZ derivatives	L-asparaginase	In vitro	Improved thermostability and proteolytic resistance without loss of activity [83]
Protein delivery	PEGylated-PCPP + spermine	Lysozyme	In vitro	High antibacterial activity; low polydispersity; membrane-disruptive effect [93]
Protein delivery	Protamine-PPZ	Exendin-4	In vivo (diabetic mouse)	Hydrogel formation at 37 °C; sustained release; improved glycemic control vs. free protein [94]

#### 4.1.1. Gene Therapy

Cationic polymers have been explored for gene delivery for more than 50 years. They spontaneously condense nucleic acids and can be modified to generate different structures and functionalities. Parameters that can be customized include the nature of the backbone and the cationic groups, charge density, pKa, molecular weight, and branching. Changes in these parameters will impact biocompatibility, biodegradability, membrane interactions, and ultimately, delivery performance [95]. Polyethyleneimine (PEI) remains the gold standard cationic polyelectrolyte when considering gene delivery applications. However, PEI has significant limitations, as it is non-biodegradable and has shown adverse effects associated with mitochondrial dysfunction [96,97]. These limitations fuel a need for new polymers to generate PEC nanocarriers with an improved efficacy/toxicity ratio for gene delivery applications.

PPZ-based PECs have shown promise as gene nanocarriers due to their ability to complex nucleic acids and their favorable pharmaceutical properties. PPZs bearing different amine side chains have been designed for controlled degradation and optimal functionality in gene delivery.

Cationized (meth)acrylate and (meth)acrylamide polymers inspired the incorporation of cationic moieties into the PPZ backbone. These include, among others, poly [2-(dimethylamine)-ethyl methacrylate], widely used as a gene delivery vector. PPZs present the additional advantage of biodegradability, and the inclusion of similar positively charged moieties within a PPZ backbone could achieve transfection/toxicity ratios comparable to those of (meth)acrylate derivatives but with a better safety profile [98]. Indeed, Luten et al. pioneered the use of PPZs for gene delivery. They synthesized two cationic PPZ derivatives bearing either 2-dimethylaminoethanol (DMAE) or 2-dimethylaminoethylamine (DMAEA), both tertiary amines. Both DMAE-PPZ and DMAEA-PPZ degraded under physiological conditions and were able to condense plasmid DNA (pDNA). The resulting PPZ-based PECs had a positive surface charge and an 80 nm size. In vitro studies showed that these DMAE/DMAEA-PPZ: pDNA PECs exhibited a superior transfection efficiency/toxicity ratio compared to commercial transfection agents [81].

Further investigations conducted by De Wolf et al. studied the DMAEA-PPZ structure to elucidate its role in the transgene performance of nucleic acid-loaded PECs. XTT cell viability assays conducted in vitro using murine neuroblastoma (neuro 2A) cells showed that the cytotoxicity of DMAEA-PPZ PECs was proportional to the molecular weight of the polymer. Moreover, the transfection efficiency of DMAEA-PPZ: pDNA PECs was dependent on the nitrogen (PPZ)/phosphorus (pDNA) ratio of the complexes, as observed in the same in vitro model. These conclusions were further confirmed in vivo. Furthermore, higher molecular weight PPZs were more toxic in an ectopic neuro 2A tumor mouse model, while low molecular weight PPZs showed greater tumor-selective gene expression [99]. In another work performed in a similar animal model, the same research group concluded that pDNA: DMAEA-PPZ PECs induced similar levels of gene expression as linear PEI but more localized to tumor tissues, while pDNA: PEI PECs led to significant expression in non-target organs [78].

Yang et al. investigated the impact of incorporating imidazole along with DMAEA into the PPZ backbone. The transfection efficiency of imidazole/DMAEA-PPZ: pDNA PECs was evaluated in vitro using three different human cell lines. Flow cytometry results showed that PECs containing imidazole had a better transfection/toxicity ratio than those based on PPZs substituted only with DMAEA. Furthermore, the overall efficacy of the system was comparable to that of PEI [100]. A decrease in toxicity with the inclusion of imidazole was also demonstrated by Ma et al. This group hypothesized that the presence of cyclic amines would be less toxic than linear or branched structures since positive charges are restricted to the heterocyclic ring. Thus, they synthesized a library of cationic PPZ polyelectrolytes for pDNA delivery grafted with cyclic polyamine and imidazole groups. In vitro results confirmed that imidazole/cyclic polyamine PPZ: pDNA PECs were less toxic to 293T cells than DMAEA-PPZ PECs [20].

Luten et al. synthesized another cationic PPZ, this time incorporating co-diamino butane (co-AB) and DMAEA, as primary and tertiary amines, respectively. (DMAEA-co-BA)-PPZ was associated with pDNA, and the resulting PECs were coated with either polyethylene glycol (PEG) or PEG-folate as a targeting moiety. Both coated and uncoated formulations had low toxicity in OVCAR 3 cell cultures. Colorimetric and confocal microscopy tests in this cell line revealed higher transfection efficiency for pDNA-loaded PECs incorporating PEG-folate than for those just PEGylated or uncoated. These results suggest effective targeting in vitro by the folate moiety. Additionally, studies with erythrocytes indicated that PEG-coated PECs induced less hemolysis compared to those lacking PEG [82].

Functionalization of cationic PPZ polyelectrolytes to target tumor sites was also explored by Yang et al. These authors synthesized 2-(2-aminoethoxyoxyethoxy)-PPZ with lactobionic acid, bearing galactose as a targeting moiety. These PECs showed a size of approximately 130 nm, low cytotoxicity, and high transfection efficiency in cell cultures. They were also tested in a BEL-7402 xenograft model, where they induced selective gene expression at the tumor compared to galactose-free systems. Altogether, these results indicated potential for targeted gene therapy with PPZ systems [84].

Hsu et al. used thiol-ene click chemistry to generate a library of cationic and anionic PPZs for gene delivery in glioblastoma models. These authors found that those PPZs with a primary amine (cysteamine-PPZ) yielded superior transfection compared to those having a tertiary amine [21]. Additionally, these authors and others studied the effect of adding diverse alkoxy carboxylate PPZs to improve the performance of cationic PPZ PECs by effectively generating zwitterionic nanosystems. Adding the anionic derivative 6-mercaptoexanoic acid improved the transfection efficacy of PECs due to its protonation at endosomal pH [21]. Similarly, another study concluded that introducing urocanic acid within a cationic PPZ structure allows for the formation of pDNA PECs with improved transfection efficiency. These systems also showed lower toxicity when compared to PEI PECs [101]. Recently, other zwitterionic PPZ nanoparticles in which the cationic PPZs had been modified with aliphatic groups showed improved gene transfer in vitro and a potent antitumoral effect in a murine xenograft glioblastoma model [22]. Indeed, these zwitterionic PPZ PECs were also loaded with therapeutic siRNA sequences, showing antitumoral effects in glioblastoma models both in vitro and in vivo after intratumoral injection [21].

In summary, PPZ flexibility allows the exploration of a broad chemical space searching for materials with optimal properties for gene delivery. Such exploration has resulted in PPZ bearing diverse types of cationic side groups, but also other anionic PPZs with transfection-enhancing characteristics, and the integration of stealth and targeting moieties.

#### 4.1.2. Vaccines

Prophylactic vaccination has advanced significantly with the introduction of genes encoding for antigens as therapeutic agents. Gene-based vaccines offer several advantages over traditional protein-based vaccines, such as antigen design flexibility and stimulation of both antibody and T-cell responses [102]. However, gene delivery encounters numerous biological barriers before reaching its target and triggering the desired immune response, including enzymatic degradation, rapid clearance, cell internalization, and endosomal degradation [103]. Moreover, gene-based vaccines still induce weaker immune responses in humans compared to viral protein subunits [102]. Consequently, antigenic proteins continue to be the most widely used agents for vaccination as they elicit both cellular and humoral immunity [104]. Still, protein subunits are safer, but less immunogenic than traditional live attenuated or inactivated vaccines, necessitating the co-administration of compounds that enhance antigen-triggered immune responses, known as adjuvants. Some adjuvants, such as CpG or monophosphoryl lipid A, enhance immune responses by mimicking pathogen-associated molecular patterns, thereby binding to specific receptors that activate innate immune pathways. Alternatively, adjuvants can function as delivery systems that present antigens in a clustered, structured manner, making them more recognizable to immune cells; this is the primary mechanism used by aluminum salts and oil-in-water emulsions [105]. While aluminum continues to be the predominant immunoadjuvant, a range of advanced systems have been included in recently licensed vaccines, such as MF59, AS01, AS03, AS04, and AF03 [106]. This trend reflects a growing interest in new adjuvant materials that elicit robust immune responses without toxicity, and that can be produced through simple, scalable processes.

Certain PPZ polyelectrolytes have been identified as promising vaccine adjuvants. Concretely, those featuring carboxylic or sulphonic groups are particularly suited for this purpose. Polyanionic PPZs can spontaneously self-assemble with positively charged antigens under physiological conditions, building PECs with adjuvant activity. These antigen-PECs have shown significant efficacy in augmenting immune responses to diverse bacterial and viral antigens across multiple animal models [77]. Thus far, the main PPZ materials explored as vaccine adjuvants are two homopolymers: poly[di(carboxylatophenoxy)PPZ] (PCPP) and [di(carboxylatoethylphenoxy)PPZ] (PCEP).

PCPP was the first PPZ-based vaccine adjuvant reported, and still the most studied. The mechanism of action of PCPP is under debate, but its immunoadjuvant activity is significantly higher than that of similar ionic polymers, such as alginic acid, poly (acrylic acid), and poly (methacrylic acid). Early studies on the molecular basis of PCPP’s immune-boosting effect have revealed a synergic activity derived from both antigen association and PCPP’s ability to establish specific interactions with immune cell receptors [16].

Firstly, PCPP-antigen association improves the immunogenicity of vaccines due to a physical mechanism. Experiments performed by asymmetric flow field-flow fractionation (AF4) revealed that PPCP-antigenic protein PECs are stabilized by a variety of non-covalent interactions such as hydrogen bonds, hydrophobic interactions, and van der Waals forces [87,107,108]. Thermostability experiments demonstrated that PCPP-adjuvanted H5N1 vaccines induce up to a 3.5-fold increase in antigen half-life, demonstrating antigen stabilization and the formation of a polymeric depot for sustained release [109].

Besides this physical effect, PCPP showed intrinsic adjuvant activity through the interaction with mannose and Toll-like receptors (TLRs). These TLRs include subtypes present on the plasma membrane (TLR4) and others present in endosomes (TLR3 and TLR9). Through these interactions, PCPP induces dendritic cell maturation and Th2 cytokine production [87,88].

PCPP adjuvant activity was assessed through its association with either inactivated X-31 influenza virions or the commercial trivalent influenza vaccine Fluzone. The immunogenicity of both variants of influenza PCPP PECs was evaluated in mice. After subcutaneous injection of X-31 virions formulated either in PBS or PCPP PECs, serum levels of IgM, IgG, and IgG1 were 10-fold higher in the animals immunized with the PCPP formulation. Similarly, Fluzone PCPP PECs elicited a greater antibody response than that induced by the commercial vaccine alone. This suggests a superior antigen presentation to immune cells when PCPP is incorporated in vaccine formulations [85]. PCPP was also studied for developing Ebola virus (EBOV) glycoprotein (GP) PECs and building a microneedle (MN) patch-based vaccine. The immune responses to the EBOV GP vaccine delivered via PCPP-based MN patches were compared with those from intramuscular (IM) injections of PCPP EVOB PECs at the same dose, along with a third group that received no adjuvanted EBOV GP. By the study’s end, the titers produced by MN-PCPP and IM-PCPP were approximately five-fold higher than those in the no-adjuvant group [89].

The most advanced PPZ vaccine based on PCPP PECs reached Phase I clinical trials (NCT00004579, ALVAC-HIV plus 160 glycoprotein, ogp160). Subjects who received ogp160-PCPP PECs showed higher T-cell proliferation than those receiving the same vaccine with alum as an adjuvant [90].

PCEP is structurally like PCPP but has longer aliphatic side groups before the terminal carboxylic acid. PCEP was evaluated together with PCPP for its intrinsic adjuvant activity and showed high affinity for the same TLRs [87]. Awate et al. studied the mechanism underlying this adjuvant activity. They observed that intramuscular injection of PCEP to mice induced a significant recruitment of diverse immune cells at both the injection site and the draining lymph nodes. These results confirmed the capacity of PCEP to induce strong local immunostimulation [110].

Functionally, PCEP and PCPP showed different immune pathways as vaccine adjuvants. Mutwiri et al. performed an immunological test in vivo with each anionic PPZ polyelectrolyte. Concretely, X-31 influenza antigen was administered in mice with different adjuvants: (i) complexed with PCPP, (ii) complexed with PCEP, and (iii) adsorbed on alum. Experimental groups receiving PCEP or PCPP PECs showed higher antibody titers than the group receiving the alum-adsorbed antigen, even at low doses. Analysis of X-31 antigen-specific cytokines revealed that alum and PCPP formulations trigger the production of IL-4, associated with Th2 immune responses. Contrarily, the PCEP formulation induced the production of both IFN-γ and IL-4, correlated with specific cellular Th1 and humoral Th2 immune responses, respectively. These results indicate that replacement of PCPP by PCEP shifts the quality of immune responses from a predominantly Th2-type to a more balanced Th1/Th2 response [111].

Another comparative study among PCPP and PCEP supports this hypothesis. Mice were immunized intraperitoneally with hepatitis C virus envelope glycoprotein E2 antigen adjuvanted with either PCPP, PCEP, Addavax, or Alhydrogel (alum). Results showed that E2 PCEP PECs display greater neutralizing antibody titers and higher serum IgG levels compared to the unadjuvanted formulation and other adjuvants. Furthermore, measurements of IgG subclasses associated with both Th1 and Th2 immune responses confirmed superior performance of PCEP in inducing hybrid immunity, in comparison to PCPP [91].

PCEP has also been explored for its potential to induce cross-protection against different strains of swine influenza. The immunogenicity of PCEP associated with the inactivated swine influenza virus (SIV) H1N1 vaccine was tested in pigs, and cross-protection was assessed by exposing vaccinated animals to the H3N2 strain. H1N1 PCEP PECs were administered intradermally twice, and 35 days after the injection, pigs were challenged with a H3N2 virus strain. Pigs vaccinated with PCEP H1N1 PECs showed high and persistent anti-H1N1 SIV-specific and neutralizing antibody levels but no H3N2 antibody titers until exposure to the virus. Therefore, PCEP failed to induce cross-protection against different virus strains. Nevertheless, ELISPOT assays from lymph node and tracheobronchial cells showed high levels of IFN-γ, IL-13, and IL-17A production, confirming PCEP’s ability to induce strong immune responses [86].

The exact reason for the differing biological behaviors of these two structurally similar PPZ polyelectrolytes, PCPP and PCEP, remains unclear. However, it is well known that the physical and biological properties of PPZs are significantly influenced by the types and proportions of side groups attached along the polymer backbone. Thus, the main difference between PCPP and PCEP biological behavior may be related to their potential endosmolytic activity, as observed in a red blood cell culture model. PCEP showed membrane-disruptive activity at early endosomal pH, while PCPP appears not to have this capability [112]. This effect could be due to the higher hydrophobicity of PCEP, which may induce membrane destabilization due to the formation of hydrophobic aggregates within the tested pH range [113]. This difference might allow PCEP to deliver part of the antigens intracellularly, thus engaging in other immune processing pathways, and generating more complex Th1/Th2 responses.

PPZ polyelectrolytes can also be combined with other polycations, such as spermine, to generate more crosslinked structures in the form of microparticles, nanoparticles, hydrogels, and microneedle arrays. For this last technological approach, PCPP was applied as a vaccine adjuvant and as a key microfabrication material to produce hepatitis B surface antigen PCPP microneedle patches. This delivery system showed superior in vivo performance in pigs with remarkably higher IgG titers than hepatitis B surface antigen PCPP PECs administered intramuscularly [114].

One last anionic PPZ polyelectrolyte developed for adjuvant purposes is poly[di(carboxylatomethylphenoxy)phosphazene] (PCMP). PCMP was rationally designed based on the knowledge accumulated from PCPP and PCEP chemical structure and their biological behavior. PCMP is structurally like PCPP, but its lateral chains have a methylene group between the aromatic ring and the carboxylic acid moieties. Moreover, the PCMP side group, 4-hydroxyphenylacetic acid, is a constituent of numerous food components and can be naturally found in a variety of human tissues and biofluids, which should ensure its biocompatibility and innocuous metabolism.

PCMP was associated with a human papillomavirus virus-like particle (HPV-VLP) vaccine, and its adjuvant potential was evaluated in vitro and in vivo, using PECs comprising PCPP and HPV-VLPs as a benchmark. PCMP demonstrated similar properties to PCPP in vitro, in terms of hydrolytic degradation and immunostimulatory properties. Furthermore, PCMP PECs showed superior stability in solutions with high ionic (sodium) content than PCPP ones. This may be due to the presence of the methylene spacer group, which gives more flexibility to side chain stereoisomerism and promotes stabilization by π–π interactions of the aromatic groups. Indeed, dynamic light scattering analysis showed that PCMP PECs display higher short-term stability than PCPP. Moreover, PCMP-adjuvanted HPV-VLP formulations induced superior neutralizing antibody responses than alum-adjuvanted HPV-VLPs and Gardasil-9 in a mouse model [92].

In summary, current studies suggest that anionic PPZ polyelectrolytes can associate with antigens and form PECs, acting as safe and effective adjuvants. Indeed, antigenic PPZ PECs can trigger both humoral and cellular immune responses against common viruses such as influenza or papillomavirus, thereby spearheading a new generation of polymer-based vaccine adjuvants. Further studies are needed to validate their efficacy, particularly in comparison to other established adjuvants, and to confirm long-term safety in human trials.

#### 4.1.3. Protein Delivery

The discovery that anionic PPZ polyelectrolytes associate with antigenic proteins and interact with cell membrane receptors raises the possibility of expanding their use to the delivery of other kinds of therapeutic proteins. Particularly, protein: PPZ PECs could overcome the short half-life and low cellular uptake of most proteins, two limitations that hinder their clinical translation. The main technological strategy used to improve protein pharmacokinetics is PEG conjugation. This conjugation increases protein molecular weight, avoiding its renal clearance. Additionally, due to its hydrophilic nature, PEG can form a steric shield around the protein that reduces its recognition by phagocytes [115]. PPZs have been proposed as alternatives to PEG conjugation for protein delivery. As an advantage, conjugation is not required in this case, as PPZs can spontaneously form PECs with proteins [83].

PPZs specifically designed for protein delivery possess anionic and neutral hydrophilic side groups. The anionic groups participate in protein complexation, whereas the neutral hydrophilic ones mimic the chemical structure of PEG. The first reported copolymer with these properties was a PPZ polyelectrolyte bearing phenoxy propionic acid (PPA) and propyl pyrrolidone (PPD) side groups, being PPA as the anionic group and PPD the neutral, hydrophilic one. Avidin was used as a model protein, and association with PPA/PPD-PPZ did not interfere with its affinity for biotin. Additionally, the cellular uptake of avidin in carcinoma Cal27 cell cultures was drastically enhanced when delivered within PPA/PPD-PPZ PECs. Hemolysis tests confirmed a membrane-disruptive effect of PPA/PPD-PPZ, which was active only under early endosome pH conditions [79].

Another pH-sensitive PPZ heteropolymer was designed by introducing either carboxylic acid or tertiary amino groups, respectively, together with PEG. These polymers were used to form PECs with L-asparaginase. AF4 characterization showed that the protein was completely bound to the PEGylated PPZ. These PECs improved the thermostability and proteolytic resistance of the loaded enzyme, while preserving its enzymatic activity [83].

PCPP derivatives have also been studied for protein delivery. Concretely, a PEGylated-PCPP derivative was synthesized and used to build PECs loaded with lysozyme, in the presence or absence of the ionic crosslinker spermine tetrahydrochloride. Those resulted in two different PEGylated PECs that were assessed for their antibacterial activity in vitro. Results demonstrated higher activity of the PEGylated PECs that were crosslinked with spermine. Crosslinked PECs were also less polydisperse and allowed protein presentation to cells. This enhanced performance of spermine-treated PECs could be attributed to a membrane-disruptive activity, opening new opportunities for protein delivery [93].

Another material was produced by conjugating protamine to the PPZ backbone (ProPPZ). The resulting polyelectrolyte ProPPZ was associated with the slightly anionic protein Exendin-4. Exendin-4: ProPPZ PECs formed nanospheres in an aqueous solution at room temperature but became hydrogels when exposed to body temperatures. The efficacy and release kinetics of Exendin-4: ProPPZ PECs were tested in a diabetic mouse model and compared to the administration of the free protein. Exendin-4: ProPPZ PECs exhibited prolonged release kinetics, and those animals treated with the protein complexed to ProPPZ showed more stable glucose levels than the controls [94].

In summary, mainly polyanionic, but also polycationic, PPZs have demonstrated the ability for protein complexation. Thereby, polyanionic PPZ heteropolymers with additional hydrophilic groups can serve as a protective, stabilizing shell similar to PEG, but without the need to establish covalent bonds with the protein. This modulation of the biopharmaceutical profile of different proteins without covalent binding could avoid the reduction in bioactivity typically observed from PEGylation.

### 4.2. Nanocarriers Based on Hydrophilic/Hydrophobic Interactions

Amphiphilic polymers are gaining attention for drug delivery due to their ability to form structured nanocarriers like micelles and polymersomes. These materials self-assemble based on their hydrophilic and hydrophobic interactions, allowing encapsulation and targeted release of water-soluble and non-polar substances [116]. Self-assembled macrostructures built from amphiphilic blocks often have controlled release properties, imparting nanocarriers with the capacity to provide a sustained therapeutic effect [117].

Many amphiphilic nanocarriers, especially diblock copolymers of polyesters, e.g., PLGA, poly(ε-caprolactone) (PLC), and PEG, offer advantages due to their regulatory profile. Nevertheless, these polymers have a rigid structure that restricts modifications for controlling release kinetics, and their degradation products increase the pH and can interact with the encapsulated drugs [27,118,119]. Other typical amphiphilic polymers are poloxamers, whose release kinetics cannot be easily modified and are non-degradable [120,121]. These limitations spark a general interest in expanding the materials toolbox available for engineering these drug nanocarriers.

**Figure 6:**
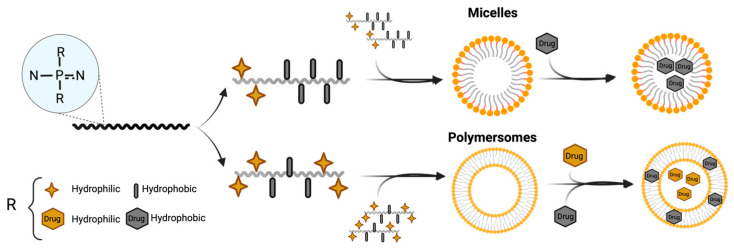
Schematic representation of the introduction of hydrophilic and hydrophobic moieties within the PPZ backbone, the subsequent macro-assembly of amphiphilic PPZs into micelles or polymersomes, and the varying water-solubility profiles of the drugs that can be encapsulated. Created in BioRender. Garcia-Fuentes, M. (2025) https://BioRender.com/lm14gpe (accessed on 29 July 2025). Based on data from references [24,26,125,126].

In this scenario, amphiphilic PPZs present enhanced drug loading and improved drug stability compared to other synthetic polymers, such as PLGA and PCL. PPZ’s unique tunable structure allows for the introduction of the optimal ratio of hydrophilic/hydrophobic side chains and the formation of stable micelles and polymersomes with greater payload capacity. Furthermore, PPZs provide benefits compared to PEG, which may lead to hypersensitivity in some patients and faster blood clearance. PPZs exhibit reduced immunogenicity compared to PEG and are safer for multiple administrations than non-biodegradable polymers [122]. PPZ’s chemical flexibility enables precise control of final polymer properties, allowing for a wide range of applications and improved performance [48]. This flexibility is perfectly illustrated in the modification of release properties, as the introduction of side groups in the PPZs can markedly change release rates, resulting in temperature-, pH-, ionic-, or redox-responsive behavior [61,66,123,124]. Indeed, amphiphilic PPZ-based micelles and polymersomes can encapsulate drugs of different solubility profiles and have shown promising potential in drug delivery and other nanomedicine applications (Figure 6, Table 3).

#### 4.2.1. Polymeric Micelles for Hydrophobic Drugs

When amphiphilic polymers possess long hydrophilic regions relative to their hydrophobic counterparts, they tend to form micelles. Polymeric micelles concentrate their hydrophobic blocks in the core, acting as a reservoir for the entrapment of poorly soluble drugs, while the system remains stabilized as an aqueous suspension by the hydrophilic shell [76]. Polymeric micelles can load a variety of hydrophobic drugs and modulate their distribution and pharmacokinetics, advantages that have facilitated their progression to clinical phases [132]. Amphiphilic PPZs have been explored to develop micelles but represent a minor yet active trend in drug delivery, since their research is limited to a few authors and some preliminary data.

##### 4.2.1.1. Anticancer Drugs

PPZ-based micelles have shown considerable promise as nanocarriers for hydrophobic anticancer drugs such as doxorubicin (Dox) and paclitaxel. These systems are built from amphiphilic PPZ copolymers that self-assemble into nanoscale micelles, enabling efficient drug encapsulation and controlled release. Early designs incorporated thermosensitive copolymers combining hydrophilic N-isopropylacrylamide with hydrophobic segments like poly(p-phenylene) or ethyl 4-aminobenzoate (EAB). These formulations demonstrated favorable self-assembly behavior, including low critical micelle concentrations and well-defined micelle sizes, as confirmed by transmission electron microscopy [25,133].

Subsequent developments introduced methoxy-polyethylene glycol (mPEG) and glycine ethyl ester (GlyEE) into the PPZ backbone. The higher hydrophilicity of mPEG facilitated micelle formation and enabled Dox encapsulation with efficiencies exceeding 80%. These micelles exhibited pH-sensitive drug release, with faster degradation and drug release under acidic conditions, like those of the tumor microenvironment. Interestingly, micelle size played a critical role in cellular uptake: smaller micelles (~147 nm) were more efficiently internalized by Adriamycin-resistant MCF-7/adr breast cancer cells than larger ones (~279 nm) [134].

Further innovations involved modifying a PCPP backbone by grafting poly (lactic acid) (PLA) to create amphiphilic copolymers. Colic acid was also introduced as a targeting ligand for the Farnesoid X receptor, commonly overexpressed in cancer cells. These micelles successfully co-encapsulated paclitaxel and indocyanine green, with drug release rates closely correlated to polymer degradation. Acidic conditions significantly accelerated hydrolysis, enhancing drug diffusion and micelle erosion [24].

In a related approach, colic acid-modified PCPP was further functionalized with poly (diallyl dimethyl) ammonium chloride (PDADM) to introduce pH-responsive behavior. These micelles exhibited a surface charge shift from negative to positive as pH decreased, promoting enhanced cellular uptake and drug release in acidic environments. In vitro studies using MCF-7 cells confirmed that these micelles improved paclitaxel delivery and cytotoxicity compared to the free drug [124].

Overall, PPZ-based micelles offer a versatile platform for solubilizing poorly water-soluble drugs, achieving controlled and targeted release, and enhancing drug efficacy through structural and functional modifications. While in vitro results are encouraging, particularly in terms of cellular uptake and cytotoxicity, in vivo studies remain scarce. Further research is needed to assess their biocompatibility, pharmacokinetics, and therapeutic efficacy in preclinical models to fully realize their potential in cancer therapy.

#### 4.2.2. Polymersomes for Hydrophilic Drugs

Alternatively, when amphiphilic polymers present larger hydrophobic domains, they self-assemble into polymersomes. Polymersomes are nanoscale vesicles, structurally like liposomes, but constituted of polymers. They form a polymeric bilayer where their hydrophilic domains protrude into the internal and external aqueous phase. Since they arrange in this form, an intralaminar, non-polar chamber is formed by the hydrophobic parts facing each other [76]. This structure is very versatile for drug delivery, as it allows for encapsulation of hydrophilic molecules in the inner aqueous reservoir and hydrophobic ones in the interlaminar chamber.

PPZs have been explored to build polymersomes and have been used mostly as vehicles for hydrophilic anticancer drugs such as the water-soluble form of doxorubicin, doxorubicin hydrochloride (Dox-HCl), or carboplatin. Additionally, and to a minor extent, they have been used for co-loading of hydrophilic and hydrophobic doxorubicin (Dox-HCl and Dox), and nucleic acid delivery.

##### 4.2.2.1. Anticancer Drugs

PPZ-based polymersomes have been extensively explored for the delivery of Dox-HCl. Early work by Zheng et al. introduced a library of amphiphilic graft PPZs bearing mPEG and EAB side chains. The self-assembly behavior of these copolymers was found to depend on the mPEG content: high mPEG ratios favored micelle formation, while lower ratios led to the formation of polymersomes. These polymersomes successfully encapsulated Dox-HCl and enhanced its antitumoral activity in HepG2 liver cancer cell cultures [126].

Building on this foundation, researchers developed pH-responsive PPZ polymersomes by incorporating diisopropylamino (DPA) groups alongside PEG chains. These DPA-PEG-PPZ copolymers formed polymersomes capable of releasing Dox-HCl selectively at acidic pH (5.5), mimicking the tumor microenvironment. Notably, polymersomes with a high DPA content inhibited the P-glycoprotein efflux pump, thereby enhancing drug retention and cytotoxicity in drug-resistant MCF-7 breast cancer cells [135,136].

**Figure 7:**
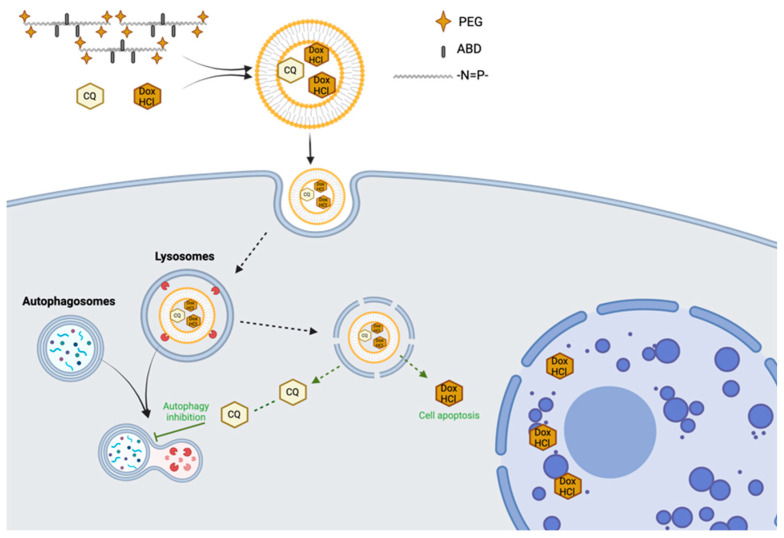
Structural composition and mechanism of action of Dox-HCl/CQ co-loaded polymersomes based on amphiphilic PPZs for tumor treatment. Created in BioRender. Garcia-Fuentes, M. (2025) https://BioRender.com/i4t52ir (accessed on 29 July 2025). Based on data from reference [127].

Further innovations included co-loading strategies to enhance therapeutic efficacy. One study used mPEG- and 4-aminomethyl-2-benzyloxy- [1,3]-dioxolan (ABD)-modified PPZs to co-encapsulate Dox-HCl and chloroquine (CQ), an autophagy inhibitor. In vivo testing in mice with drug-resistant K562/ADR tumors showed that this combination prolonged drug circulation and significantly improved antitumoral effects, likely due to CQ’s ability to sensitize cancer cells by inhibiting autophagy (Figure 7) [127].

The dual-loading capacity of PPZ polymersomes was further demonstrated by Khan et al., who developed dual-responsive polymersomes capable of encapsulating both hydrophilic Dox-HCl and hydrophobic Dox. These polymersomes were made of a reductive/acidic dual-responsive PPZ bearing mPEG and N, N-Diisopropylethylenediamino (DPEA). These mPEG-DPEA-PPZ polymersomes achieved high encapsulation efficiency (~90%) and showed pH-dependent release, with faster drug release under acidic conditions [26].

Finally, Xu et al. systematically investigated how the hydrophilic/hydrophobic balance in PPZ side chains affects drug encapsulation and delivery. They confirmed that higher mPEG content favors micelle formation, while higher EAB content supports polymersome assembly. Both Dox-HCl and Dox were efficiently encapsulated in polymersomes, which outperformed micelles in terms of cellular uptake and therapeutic efficacy in MCF-7 xenograft models. Importantly, polymersomes reduced systemic toxicity without compromising antitumoral activity [137].

To improve structural stability, Fu et al. developed hybrid mPEG-EAB PPZ polymersomes incorporating gold nanoparticles (AuNPs). These AuNPs acted as cross-linkers within the EAB-based bilayer, stabilizing the polymersome structure and preventing premature drug leakage at physiological pH. The resulting formulation maintained the pH-responsive release and demonstrated improved therapeutic outcomes in a murine sarcoma xenograft model [28].

Targeted delivery strategies were also explored. Li et al. functionalized similar mPEG-EAB-PPZ polymersomes with the AS1411 aptamer, which binds nucleolin, a protein overexpressed in many cancer cells. The aptamer was conjugated to cholesterol and inserted into the hydrophobic bilayer of the polymersomes. These modified carriers showed high Dox-HCl loading capacity and significantly improved antiproliferative effects in MCF-7 cells. In vivo studies in mice bearing MCF-7 tumors confirmed enhanced drug accumulation and superior antitumoral efficacy compared to free Dox-HCl [128].

Of note, PPZ polymersomes have also been adapted for the delivery of other hydrophilic drugs, such as carboplatin. Zhu et al. synthesized amino-functionalized mPEG-EAB-PPZ copolymers using tris(2-aminoethyl) amine to enhance carboplatin loading. These formulations showed no toxicity in blank form and demonstrated superior antiproliferative activity and tumor targeting compared to free carboplatin in CT-26 colon adenocarcinoma cells [129].

##### 4.2.2.2. Nucleic Acids

The use of PPZ polymersomes for nucleic acid delivery has not yet been extensively studied. Nevertheless, their structure could be useful for incorporating nucleic acids into the internal cavity, and some authors have described interesting findings in this area. Peng et al. prepared miRNA PPZ polymersomes by using two amphiphilic PPZs: mPEG-EAB-PPZ and mPEG-DPA-PPZ. The polymersomes were loaded efficiently with the antitumoral sequence miR-200c. Even at low doses, miR-200c PPZ polymersomes showed powerful antiproliferative and pro-apoptotic activity in human lung cancer (A549/T) cell cultures and in xenograft tumors implanted in mice [130].

PPZ polymersomes have also been loaded with pDNA. Concretely, DPEA-mPEG PPZ polymersomes were developed by Gao et al. to deliver IL-12 plasmid (pIL12). Toxicity and cellular uptake were assessed in B16 and CT-26 cell cultures, and results showed good cell tolerance of the formulation. In addition, pIL12 loaded in PPZ polymersomes showed higher internalization than the naked plasmid. These PPZ polymersomes were administered by intravenous bolus to mice bearing CT-26 colon carcinoma, resulting in significant suppression of tumor growth [131].

### 4.3. Polymer–Drug Conjugates

Polymer–drug conjugates represent a well-studied strategy in drug delivery, where a polymeric backbone acts as a carrier, and bioactive agents are chemically bound by a labile linker. This flexible connector can release the active molecules under specific conditions, providing tissue selectivity and targeting properties. Ultimately, the polymer’s physicochemical characteristics will determine the resulting conjugate’s biopharmaceutical properties [138].

**Figure 8:**
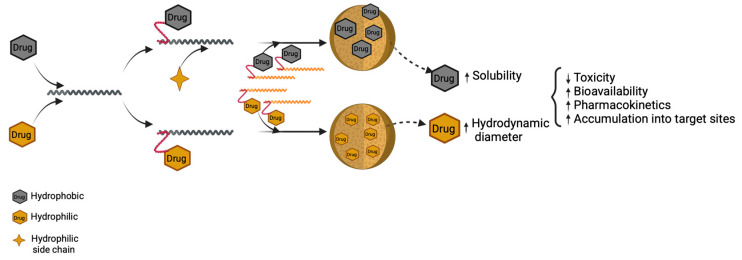
Schematic representation of PPZ–drug conjugation strategies, including optional side chain further incorporation, to optimize solubility (grey symbol- hydrophobic drugs) or molecular weight (yellow symbol- hydrophilic small drugs) for improved drug biopharmaceutical properties. Created in BioRender. Garcia-Fuentes, M. (2025) https://BioRender.com/1623r8s (accessed on 29 July 2025).

**Figure 9:**
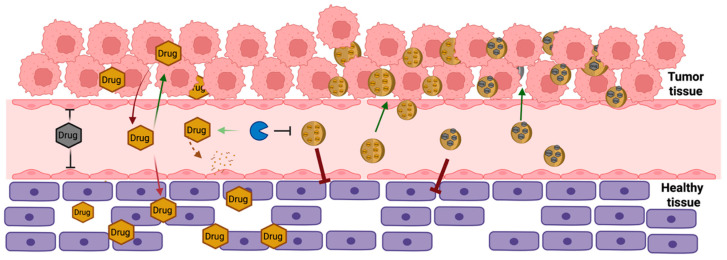
Schematic comparison of the different pharmacokinetic behavior of small hydrophilic (yellow) or hydrophobic (grey) drug molecules and the benefits resulting from their conjugation to polymers in their biodistribution, limited in healthy tissues (red arrow), while increased accumulation into tumor tissues (green arrow). Created in BioRender. Garcia-Fuentes, M. (2025) https://BioRender.com/5rdn0yh (accessed on 29 July 2025).

The main advantages resulting from drug conjugation are improved drug solubility and reduced toxicity. One key advantage of drug–polymer conjugates is the increase in the hydrodynamic diameter in the case of hydrophilic drugs or modifying the solubility profile for hydrophobic ones. The size increase extends the drug’s plasmatic circulation time, thus changing the pharmacokinetic profile of the compound. Moreover, polymer–drug conjugation protects therapeutic agents from enzymatic degradation and favors drug accumulation at the target sites (Figure 8 and Figure 9) [139,140].

Among the variety of available biodegradable polymers, PPZs represent a unique structure. The chlorine atoms of the PDCP precursor can be readily substituted by active molecules. In this scenario, polymer conjugates can be prepared straight from this precursor. On this basis, a few prototypes of PPZ–drug conjugates have been prepared, mostly for the delivery of anticancer drugs (Table 4).

#### 4.3.1. Anticancer Drugs

Platinum-based chemotherapeutics such as cisplatin, carboplatin, and oxaliplatin remain central to cancer treatment, yet their clinical utility is often compromised by systemic toxicity, poor tumor selectivity, and the development of drug resistance. To overcome these limitations, polymer–drug conjugation has emerged as a promising strategy, offering controlled drug release and the potential for passive tumor targeting [143].

Among the various polymer platforms explored, PPZs have shown potential for platinum Pt (II) drug delivery. Early work by Sohn et al. synthesized PPZ-(diamine) Pt (II) conjugates, using dicarboxylic amino acids as spacers. These conjugates exhibited potent cytotoxicity across multiple human cancer cell lines and in murine leukemia models, frequently outperforming free cisplatin. Notably, the glutamate-linked PPZ conjugate demonstrated the highest activity and showed no cross-resistance to cisplatin [144].

**Figure 10:**
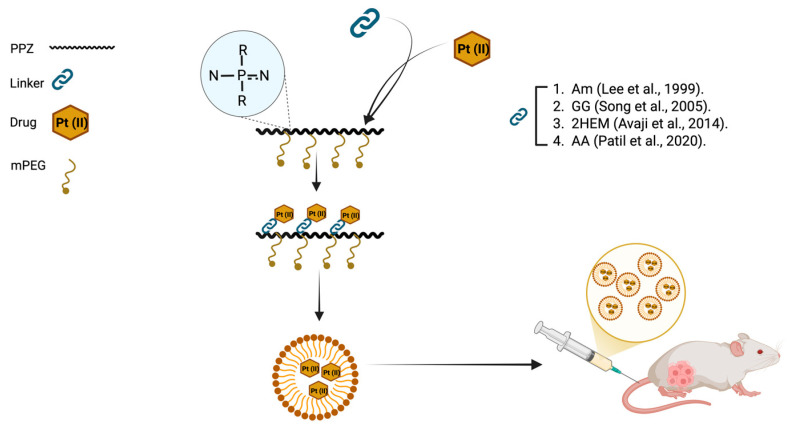
Representative image of the Pt (II) conjugation strategy with mPEG PPZ using a variable linker in the various studies performed over 20 years. Created in BioRender. Garcia-Fuentes, M. (2025) https://BioRender.com/vplc111 (accessed on 29 July 2025). Based on data from the publications cited in the illustration [29,31,145,146].

Building on this foundation, subsequent studies incorporated mPEG into the PPZ backbone to improve solubility and enable pH- and temperature-sensitive hydrolytic degradation. mPEG-PPZ-Pt (II) conjugates exhibited enhanced antitumor activity in vivo, attributed to their ability to release the drug in a controlled manner. Further refinements on these mPEG-PPZ-Pt (II) conjugates focused on optimizing the spacer chemistry. Platinum was conjugated via glycyl-L-glutamate (GG), 2-hydroxyethoxydiethylmalate (2HEM), or cis-aconitic anhydride (AA). The resulting amphiphilic mPEG-PPZ-Pt (II) conjugates self-assembled into micelles ranging from 18.6 to 130 nm (Figure 10). These systems retained cytotoxicity comparable to free drugs in vitro. In preclinical models, GG-linked micelles showed improved tumor accumulation and extended circulation time, while 2HEM-linked conjugates demonstrated superior tumor selectivity and reduced renal toxicity. Although AA-linked conjugates were less effective at low doses, they outperformed free drugs at higher concentrations. Across all formulations, nephrotoxicity was significantly reduced, even at double the dose of free cisplatin [29,31,145,146].

The development of macromolecular prodrugs through the conjugation of Pt (IV) complexes to PPZs introduced another innovative approach. These macromolecular conjugates, designed to undergo intracellular reduction to active Pt (II) species and simultaneous release from the polymer carrier, exhibited markedly enhanced cellular uptake and cytotoxicity in cell cultures. They also showed reduced resistance in drug-resistant cell lines. However, in vivo efficacy in CT-26 tumor models was modest, suggesting further optimization is required [141].

Expanding the scope of metal-based therapeutics, Hack et al. conjugated ruthenium and rhodium complexes to highly branched PPZs. These conjugates, synthesized using Jeffamine and protected diamine side chains, demonstrated promising antitumor activity in CT-26 colon carcinoma models. Rhodium-PPZ conjugates, in particular, significantly extended survival and reduced local toxicity [62].

Beyond platinum-based agents, PPZs have also been employed to deliver other chemotherapeutics. Teasdale et al. developed PPZ-epirubicin conjugates functionalized with folic acid for tumor targeting and acid-sensitive linkers for controlled release. These systems released epirubicin preferentially under acidic conditions, mimicking the tumor microenvironment, and their degradation rate could be tuned by modifying PPZ side chains [147].

The versatility of the PPZ platform was further demonstrated by conjugating docetaxel via a lysine ethyl ester linker to mPEG-PPZ. These conjugates formed ~40 nm micelles and, when labeled with a cyanine dye, exhibited prolonged drug circulation and selective tumor accumulation. In murine models, they achieved complete tumor regression with minimal systemic toxicity [30].

#### 4.3.2. Other Drugs

Kumar et al. developed antibiotic-PPZs conjugates as a new combination regimen for the treatment of resistant malaria. PPZs with the following side chains were synthesized: 2-propoxy, 4-acetamidophenoxy, 4-formyl, and 4-aminoethylbenzoate. These PPZs were subsequently conjugated to the antimalarial drugs dihydroartemisinin and primaquine. The presence of hydrophilic and hydrophobic side groups led to micellar antibiotic-PPZ conjugates. The conjugates were evaluated for antiplasmodial activity in *Plasmodium berghei*-infected mice. PPZ conjugates showed greater antimalarial effect than the standard line of treatment, even at low doses. This conjugation of dihydroartemisin and primaquine to PPZs also resulted in sustained protection for over 35 days without recurrence, proving not only its efficacy but also its safety. Such results raise interest in further research and highlight the potential for clinical development of the formulation [142].

In summary, most research on PPZ conjugation focuses on platinum (Pt (II) and Pt (IV)) and other chemotherapeutics, such as docetaxel and epirubicin. These drugs have been covalently attached to PPZs using various biodegradable, pH-sensitive, tumor-targeting linkers. As a result, these conjugates often self-assemble into macrostructures, such as micelles, which improve tumor accumulation, reduce systemic toxicity, and, in some cases, outperform free drugs in preclinical models. Additionally, PPZ conjugates have been investigated for delivering ruthenium, rhodium, and even antimalarial drugs, demonstrating enhanced efficacy and safety profiles across various disease models.

## 5. Closing Perspective

PPZs are a class of synthetic polymers with a long-standing history in biomedical research. Although their clinical application has not progressed as steadily as other polymeric materials, their exceptional chemical versatility and functional adaptability position them as highly promising candidates for a wide range of nanotherapeutic applications. Unlike many other materials, PPZs can be engineered to form virtually all major nanomedicine architectures, including polyelectrolyte complexes (both cationic and anionic), polymeric micelles, polymersomes, and polymer–drug conjugates [6,7,8]. This structural and functional diversity is rare and underscores the broad potential of PPZs in advanced drug delivery systems.

Currently, the most clinically advanced PPZ-based nanotherapeutics are found in the field of nanovaccines, with some formulations already undergoing early-phase clinical trials [77,90]. These developments pave the way for the future use of cationic PPZs in gene delivery, potentially offering more effective alternatives to lipid-based nanoparticles [21,82,84]. Moreover, the ability of PPZs to form targeted delivery systems—such as polymersomes, nanomicelles, and drug conjugates—holds great promise for site-specific drug delivery, particularly in oncology [28,31,127,129,135,143].

To fully realize the potential of PPZs in nanomedicine, future research must embrace a more multidisciplinary approach. This includes fostering collaboration between synthetic chemists capable of designing complex PPZ architectures and pharmaceutical scientists who can guide the rational design of formulations based on biopharmaceutical requirements. A deeper understanding of the supramolecular structures formed by PPZs will be essential to optimize their performance and tailor their behavior for specific therapeutic goals.

Equally important is the consideration of regulatory and translational aspects. As PPZ-based systems advance toward clinical application, attention must be given to scalable manufacturing under GMP conditions, regulatory compliance, and integration with clinical development pathways. The recent FDA approval of the COBRA-PzF™ coronary stent, which features a PPZ nanocoating, marks a significant milestone [10]. This approval not only validates the clinical safety of the PPZ backbone but also opens the door for accelerated regulatory pathways for other PPZ-based nanotherapeutics.

Despite their promising versatility, using PPZs in nanotherapeutics still presents challenges such as reproducible large-scale synthesis, stability in physiological environments, and regulatory hurdles stemming from their synthetic origin. Notably, recent research has investigated PPZs in sectors such as cosmetics for safer and more effective UV filters and as antibacterial coatings for medical devices [148,149,150]. These cross-sectoral uses demonstrate the flexibility of PPZs and provide important lessons for improving their design and gaining regulatory approval for advanced biomedical applications.

In conclusion, polyphosphazenes offer a unique and powerful platform for the development of next-generation nanomedicines. Their multifunctionality, biodegradability, and tunable properties make them ideal candidates for addressing current challenges in drug delivery, gene therapy, and immunotherapy. With continued interdisciplinary collaboration and strategic clinical translation, PPZs are poised to play a central role in the future of nanotherapeutics.

## Figures and Tables

**Table 1:** Summary of different PPZs categorized by their side chains and their degradation products.

PPZ Type	Side Group	Degradation Products		Ref.
Amino-PPZ	Imidazole	Amino acid	+ Phosphate + Ammonia	[53,54]
	Amino acid ester	Amino acid/alcohol		[53,54]
Alkoxy-PPZ	Glyceryl	Glycerol		[55]
	Glucosyl	Glucose		[56]
	Methyl amino	Methylamine		[56]
	Glycolic acid ester	Glycolic acid Benzyl alcohol/ethanol		[57]
	Lactic acid ester	Lactic acid Benzyl alcohol/ethanol		[57]

**Table 3:** PPZ-based nanocarriers constituted of hydrophilic and hydrophobic interactions. Compilation of experimental studies from the last 10 years (2015–2025) based on the use of amphiphilic PPZ for the delivery of drugs of different solubility profiles.

Therapeutic Application	PPZ Derivative	Cargo	In Vitro/In Vivo Model	Main Finding and Ref.
Hydrophobic anticancer drug delivery	Colic acid-PLA- PCPP	Paclitaxel/indocyanine green	In vitro (MCF-7)	Sustained release; accelerated in acidic cancerous conditions [24]
Hydrophobic anticancer drug delivery	Colic acid-PDADM-PCPP	Paclitaxel	In vitro (MCF-7)	Improved drug delivery and cytotoxicity vs. free drug [124]
Hydrophilic anticancer drug delivery	mPEG-ABD-PPZ	Dox-HCl/CQ	In vivo (K562/ADR)	Prolonged circulation and greater antitumor effects [127]
Hydrophilic anticancer drug delivery	mPEG-EAB-PPZ + AuNPs	Dox-HCl	In vitro (S180) In vivo (S180)	pH-responsive release; greater antitumor effects than free drug [28]
Hydrophilic anticancer drug delivery	mPEG-EAB-PPZ + AS1411	Dox-HCl	In vitro (MCF-7) In vivo (MCF-7)	Greater antiproliferative and antitumoral effect than free drug [128]
Hydrophilic anticancer drug delivery	mPEG-EAB-PPZ + tris(2-aminoethyl) amine	Carboplatin	In vitro (CT-26) In vivo (CT-26)	Greater antiproliferative activity and tumor targeting than free drug [129]
Gene delivery	mPEG-DPA-PPZ	miR-200c	In vitro (A549/T) In vivo (A549/T)	Antiproliferative and pro-apoptotic activity [130]
Gene delivery	DPEA-mPEG-PPZ	pIL12	In vitro (B6/CT-26) In vivo (CT-26)	Good tolerance, high internalization and tumor suppression [131]

**Table 4:** PPZ-based nanocarriers based on polymer-to-drug conjugation. Compilation of experimental studies from the last 10 years (2015–2025) based on drug conjugation to PPZ for the delivery of small drugs with poor pharmacokinetics.

Therapeutic Application	PPZ Derivative	Cargo	In Vitro/In Vivo Model	Main Finding and Ref.
Anticancer drugs	mPEG-PPZ	Pt (II)	In vivo (MKN-28)	Greater antitumor effect and lower cytotoxicity [31]
Anticancer drugs	Jeffamine-PPZ	Pt (IV)	In vitro (A2780/HCT116) In vivo (CT-26)	Enhanced cellular uptake and cytotoxicity; modest in vivo efficacy [141]
Anticancer drugs	Jeffamine-PPZ	Rhodium	In vivo (CT-26)	Extended survival and reduced local toxicity [62]
Anticancer drugs	mPEG-PPZ	Docetaxel	In vitro In vivo (MKN-28)	Tumor regression with minimal systemic toxicity [30]
Antibiotics	Highly branched PPZ	Dihydroartemisinin/ primaquine	In vivo (*Plasmodium berghei*-infected mice)	Sustained drug release for 35 days; greater antimalarial effect [142]

## Data Availability

No new data were created or analyzed in this study. Data sharing is not applicable to this article.

## Abbreviations

The following abbreviations are used in this manuscript:

AA	Cis-aconitic anhydride.
ABD	4-aminomethyl-2-benzyloxy-[1,3]-dioxolan
AF4	Asymmetric flow field-flow fractionation
ALVAC-HIV	Canarypox virus vector vaccine for HIV
AS1411	Nucleolin-binding aptamer
AuNPs	Gold nanoparticles
BEL-7402	Hepatocellular carcinoma cell line (human)
CG	Cytosine–phosphate–Guanine
CQ	Chloroquine
CT-26	Colon carcinoma cell line (mouse)
DMAE	2-Dimethylaminoethanol
DMAEA	2-Dimethylaminoethylamine
DPA	Diisopropylamino
DPEA	N, N-Diisopropylethylenediamino
Dox	Doxorubicin
Dox-HCl	Doxorubicin hydrochloride
EAB	Ethyl 4-aminobenzoate
EBOV	Ebola virus
GG	Glycyl-L-glutamate
GlyEE	Glycine ethyl ester
GP	Glycoprotein
H1N1	A subtype of the Influenza A virus (swine flu)
H3N2	A subtype of the Influenza A virus
H5N1	A subtype of Influenza A virus (avian influenza)
HCCP	Hexachlorocyclotriphosphazene
HepG2	Liver cancer cell line (human)
HPV-VLP	Human papillomavirus virus-like particle
IFN-γ	Interferon gamma
IgM/IgG/IgG1	Immunoglobulin M/G/G1
IL-4	Interleukin 4
LCST	Lower critical solution temperature
MCF-7	Breast cancer cell line (human)
MCF-7/adr	Adriamycin-resistant MCF-7 breast cancer cell line
mPEG	Methoxy-polyethylene glycol
OVCAR 3	Ovarian cancer cell line 3
PCPP	Poly[di(carboxylatophenoxy)phosphazene]
PCEP	Poly[di(carboxylatoethylphenoxy)phosphazene]
PBS	Phosphate-buffered saline
P-C	Phosphorus-Carbon (bond)
PDADM	Poly (diallyl dimethyl) ammonium chloride
PDI	Polydispersity Index
PEC(s)	Polyelectrolytic complex(es)
PEG	Polyethylene glycol
PEG-folate	Polyethylene Glycol conjugated with Folate
PEGylated	Modified with Polyethylene Glycol
PEI	Polyethyleneimine
PLA	Poly (lactic acid)
PLGA	Poly (lactic-co-glycolic acid)
PLC	Poly(ε-caprolactone)
pDNA	Plasmid DNA
pIL12	IL-12 plasmid
PPD	Propyl pyrrolidone
PPA	Phenoxy propionic acid
PPZ(s)	Poly(organo)phosphazene(s)
ProPPZ	Protamine-conjugated polyphosphazene
P-N	Phosphorus–Nitrogen (bond)
P-O	Phosphorus–Oxygen (bond)
SIV	Swine influenza virus
Si-N	Silicon–Nitrogen (bond)
siRNA	Small Interfering RNA
THF	Tetrahydrofuran
Th1/Th2	T helper type 1/type 2
TLR(s)	Toll-like receptor(s)
TLR7/8	Toll-Like Receptors 7 and 8
TROP	Thermal ring-opening polymerization
XTT	3-[4,5-dimethylthiazol-2-yl]-2,5-diphenyltetrazolium bromide
Pt (II)	Platinum in oxidation state +2
Pt (IV)	Platinum in oxidation state +4
